# Estimating individual trajectories of structural and cognitive decline in mild cognitive impairment for early prediction of progression to dementia of the Alzheimer’s type

**DOI:** 10.1038/s41598-024-63301-7

**Published:** 2024-06-05

**Authors:** Shreya K. Rajagopal, Adriene M. Beltz, Benjamin M. Hampstead, Thad A. Polk

**Affiliations:** 1 Department of Psychology, University of Michigan, Ann Arbor, MI USA; 2 Department of Psychiatry, University of Michigan, Ann Arbor, MI USA; 3 VA Ann Arbor Healthcare System, Ann Arbor, MI USA

**Keywords:** Alzheimer's disease, Cognitive ageing

## Abstract

Only a third of individuals with mild cognitive impairment (MCI) progress to dementia of the Alzheimer’s type (DAT). Identifying biomarkers that distinguish individuals with MCI who will progress to DAT (MCI-Converters) from those who will not (MCI-Non-Converters) remains a key challenge in the field. In our study, we evaluate whether the individual rates of loss of volumes of the Hippocampus and entorhinal cortex (EC) with age in the MCI stage can predict progression to DAT. Using data from 758 MCI patients in the Alzheimer’s Disease Neuroimaging Database, we employ Linear Mixed Effects (LME) models to estimate individual trajectories of regional brain volume loss over 12 years on average. Our approach involves three key analyses: (1) mapping age-related volume loss trajectories in MCI-Converters and Non-Converters, (2) using logistic regression to predict progression to DAT based on individual rates of hippocampal and EC volume loss, and (3) examining the relationship between individual estimates of these volumetric changes and cognitive decline across different cognitive functions—episodic memory, visuospatial processing, and executive function. We find that the loss of Hippocampal volume is significantly more rapid in MCI-Converters than Non-Converters, but find no such difference in EC volumes. We also find that the rate of hippocampal volume loss in the MCI stage is a significant predictor of conversion to DAT, while the rate of volume loss in the EC and other additional regions is not. Finally, individual estimates of rates of regional volume loss in both the Hippocampus and EC, and other additional regions, correlate strongly with individual rates of cognitive decline. Across all analyses, we find significant individual variation in the initial volumes and the rates of changes in volume with age in individuals with MCI. This study highlights the importance of personalized approaches in predicting AD progression, offering insights for future research and intervention strategies.

## Introduction

Alzheimer’s disease (AD) is the most common cause of dementia in older adults^1^. While AD is characterized by pathological changes to the brain, dementia of the Alzheimer’s type (DAT) describes a clinical phenotype in the form of an observable decline in memory and other cognitive functions. Although there are no known disease-modifying interventions that can reverse AD-based pathological changes once they have occurred, there is some evidence that modifying exposure to risk factors and incorporating lifestyle changes at an early stage of disease progression might delay the associated cognitive decline^2^. An early diagnosis also allows individuals and their caregivers time to prepare for the future.

Mild cognitive impairment (MCI) is a clinical intermediate between normal cognition (CN) and DAT, wherein patients present with cognitive decline that is greater than normal for their age and historical functioning but that does not prohibit functional independence^3,4^. However, MCI is not always a precursor to DAT. The Alzheimer’s Association reports that only a third of individuals with MCI progress to DAT within 5 years^5^. Identifying biomarkers that predict this progression at the MCI stage could be pivotal in delaying the onset of DAT symptoms in the future by allowing for the incorporation of time-sensitive interventions.

Given their lack of invasiveness, a number of MRI measures have historically been explored as biomarkers of AD, focusing on pathological structural changes in the brain. For instance, gray matter loss has been shown to begin in the temporal and limbic cortices and to spread into frontal and occipital regions over time, with much less involvement of sensorimotor regions^6^. This pattern follows the well-established Braak stages of neurofibrillary tangles forming in the brains of individuals with AD^7^. These MRI measures have also been examined as biomarkers of DAT, focusing on changes in memory and cognitive abilities. The volumes of the Entorhinal cortex (EC) and Hippocampus have historically been shown to distinguish between participants with normal cognition and DAT^8–10^. Furthermore, patients in the MCI stage exhibit intermediate levels of EC and Hippocampal atrophy^8,11^.

Given the progressive pattern of volume loss across the clinical stages of AD, the absolute volumes of the EC and Hippocampus—and seemingly their rates of decline over time—hold great potential in predicting progression to DAT during the MCI stage, but volumetric changes over disease progression remain relatively unclear. Historically, studies looking at structural biomarkers predicting progression to DAT have largely been affected by three major issues. Most studies were cross-sectional, and were not able to leverage longitudinal data to assess change (*Issue 1—Cross-Sectional Nature*). Indeed, only 48 multiple time point studies were included in a meta-analysis of Alzheimer’s biomarkers from 1995 to 2015^12^. Of these, over 50% had only one follow-up visit. These two-point studies analyze change by means of difference scores, which do not validly or reliably reflect true change^13^.

The second major issue was that sample sizes in these studies were typically relatively small (*Issue 2—Small Sample Sizes*). Of the studies included in the meta-analysis^12^, over a third had fewer than 100 participants. To add to this, these studies likely used small samples to look for small effects, as the average follow-up length for most studies in this survey ranged from 1 to 2.5 years, which is a relatively short span to see marked changes in brain volume. For reference, a two-time point study with 80% power to detect an effect with a small effect size (*d* = 0.2) would require at least 199 participants. In addition to an increased chance of detecting an effect when it exists, large sample sizes are also helpful in producing reproducible results, especially when linking inter-individual differences in structural MRI measures to cognitive performance and behavior^14^.

The third issue was the lack of incorporation of individual differences (*Issue 3—Missing Individual Differences*). Not all individuals within a diagnostic group will exhibit similar patterns of structural changes in the brain over time. In our two groups of interest—individuals with MCI who progress to DAT (MCI-Converters) and those who do not (MCI-Non Converters)—not only might the patterns of structural decline with age vary among individuals within each group, but they might even overlap with those of individuals from the other group. The concept of cognitive reserve highlights this particularly well—two individuals with the same structural changes in the brain can display very different degrees of cognitive deficit, with a greater cognitive reserve providing greater resilience against structural deterioration^15^. The strength of the cognitive reserve depends on individual factors like genetic predisposition to disease, socioeconomic status, education level, occupational attainment, and other lifestyle factors^16,17^. A group-averaged estimate of how brain structure changes over time for a particular diagnostic group is therefore unlikely to be representative of all individuals within that group and will therefore have limited utility in individually-tailored interventions.

More recent studies have overcome some of these limitations, and provide promising results. For example, there are several studies that have leveraged the power of deep learning to identify biomarkers from MRI images that can distinguish between MCI-Converters and Non Converters in the MCI stage with high accuracy^18–21^. Since the performance of deep learning models tends to rely on the size of the datasets they are trained on, this approach automatically addresses *Issue 2.* These models often leverage large datasets, and sometimes include multiple datasets to increase generalizability. They also tend to result in very high accuracy classifications provided that they are trained on large and representative data samples. Additionally, these models provide individual-specific predictions that speak to *Issue 3.* However, they also present certain challenges. Firstly, they are often purely data-driven, with feature selection in that is atheoretical and works simply by minimizing error. This can result in classifying features that do not reflect neural processes of interest and hard to interpret. Another broad concern with deep learning based models is their tendency to overfit the training data. Finally, these approaches generally struggle to accommodate dependent (e.g., longitudinal) data as they tend to use only the baseline MRI scans pertaining to each subject in their analysis (*Issue 1*).

Several other kinds of machine learning methods have also been successful in accurately predicting progression to DAT in the MCI stage^22–25^. Most machine learning methods also rely on large datasets to identify meaningful patterns, thereby addressing *Issue 2*.

The ability to address *Issues 1* and *3* depends on the specific algorithm selected. For example, Maheux et al.^26^ deploy a statistical model called AD Course Map that leverages multimodal longitudinal patient data to forecast individual disease progression trajectories. Lim and Schaar^27^ use a deep-learning based joint modeling approach to predict time of conversion to DAT and values of other longitudinal measures for individual participants. Both these studies address *Issues 1* and *3*.

However, as models become more complex and begin to yield highly accurate predictions, there is one final consideration to be made—the accuracy vs. interpretability tradeoff. A review comparing machine learning methods in predicting MCI progression made two important conclusions: (1) Adding features from multiple modalities (structural and functional MRI features, cognitive scores, demographic features, genetic information etc.) improves classification accuracies, (2) Combining multiple algorithms tends to improve classification accuracies^28^. As newer models begin incorporating multimodal inputs and combining multiple machine learning algorithms, it is important to note that higher accuracy typically comes at the cost of how well we can interpret the model’s findings, and what insights into disease progression we can draw from the results. Thus, machine learning methods are useful, and yet, would benefit from complementary approaches that more permit a prior knowledge incorporation and individual-level predictions to future timepoints and different modalities.

In our study, we attempt to address *Issues 1 to 3* within such a parsimonious model framework. Specifically, we estimate both group-averaged and individual-specific baseline regional brain volumes and their rates of change over time in the MCI stage. These specific regional brain volumes are derived from structural MRI scans of individuals with MCI. We also estimate group-averaged and individual-specific baseline performances across 3 cognitive processes—episodic memory, visuospatial processing and executive function—and their rates of change over time in the MCI stage. We evaluate if these individual specific rates of structural decline are predictive of progression to DAT in the MCI stage, and also explore whether the rates of structural decline in individuals is correlated to their rates of cognitive decline. We make use of linear mixed effects models (LME) for our analyses to address *Issues 1 and 3*. These models can yield both a group-averaged effect of a time-varying predictor on the outcome (fixed effect), and individual specific estimates for how much each individual deviates from the group-average (random effects). Additionally, these models have several advantages over more conventional methods used in longitudinal data analysis (e.g., repeated measures ANOVA), as they can handle missing data, different start and end points in the study, different durations between consequent data points, and a different number of data points per participant. This allows us to retain participants that might have been systematically excluded in more constrained analysis methods, and thus, to increase the generalizability of our findings.

We hypothesize that the rates of decline in regional brain volumes will be associated with the rate at which cognitive function deteriorates. This hypothesis builds on the ability of these brain volumes to differentiate stages of cognitive health—CN, MCI, and DAT. Our objective is to determine whether changes in regional brain volumes align with the decline in specific cognitive abilities, thereby linking the structural changes in the brain with clinical symptoms. While previous studies^29,30^ have associated Hippocampal and EC volumes with episodic memory and measures of global cognition, the relationship between the rate at which the structure of the brain changes and the rate of cognitive decline over time at an individual level in the MCI stage, remains unexplored.

To address *Issue 2*, we analyzed longitudinal structural MRI data from a large sample of 758 patients with MCI from the Alzheimer’s Disease Neuroimaging Database (ADNI) with repeated semi-annual or annual (dependent on ADNI cohort) follow-up visits per participant (followup visits up to 15 years from baseline). We address 3 main questions:

- Research Question A**:** Do MCI-Converters experience a more accelerated loss of regional brain volume with age in the Hippocampus, the EC compared to MCI-Non-Converters? Are there significant individual differences in these rates of volume loss across individuals with MCI?
- Research Question B**:** Can individual rates of loss of volume in the Hippocampus and EC in the MCI stage predict progression to DAT?
- Research Question C**:** Are individual rates of loss of volume in the Hippocampus and EC correlated with the individual rates at which cognitive abilities decline in individuals with MCI?

In addition to the regional brain volumes of the Hippocampus and the EC, we also evaluate these questions in a set of additional regions—Lateral Ventricles, Occipital Lobe, Fusiform Gyrus, and the Whole Brain. We evaluate the Lateral Ventricles as another possible region of interest in disease progression, given previous research indicating its potential as a biomarker for progression to DAT^31–33^. The Occipital Lobe and Fusiform Gyrus are selected as control regions since they are largely unaffected in the early stages of disease progression^34^. Evaluating the Whole Brain volume serves to ensure that any region-specific effects we observe are not simply the result of age-related volume loss of the brain as a whole.

## Results

### LME models of regional brain volumes in the MCI stage

#### Hippocampal and entorhinal cortex trajectories

An LME model was used to examine the effect of age, cohort, and their interaction on the standardized Hippocampal volume of participants with MCI. Data from visits after conversion to DAT were excluded for converters in order to test if pre-conversion data could distinguish converters from non-converters.

**Figure 1:**
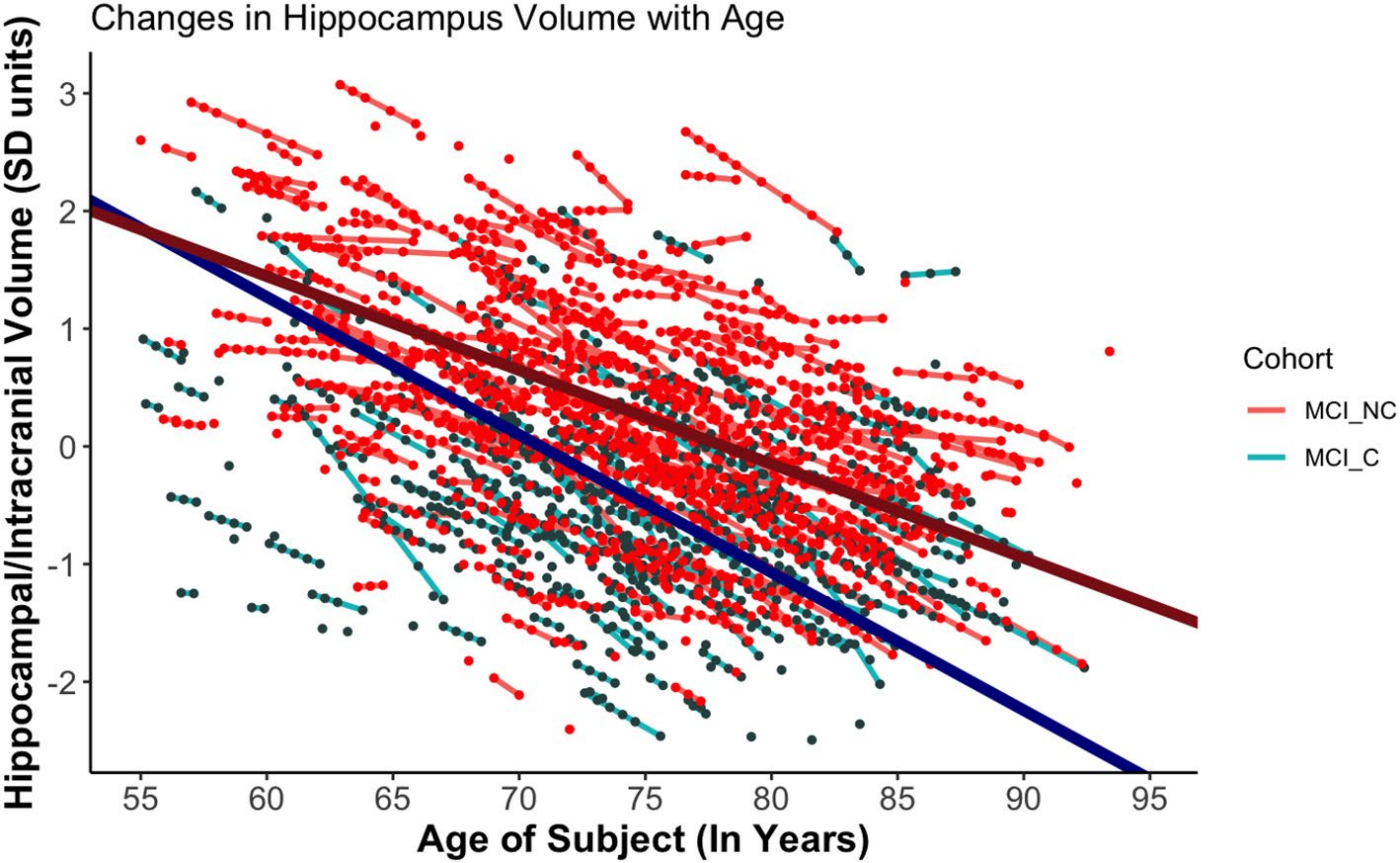
Trajectories of Hippocampal Volume Loss with Age. The trend lines represent the average trajectory of loss of standardized Hippocampal volume with age for each MCI Cohort (Dark red = MCI-Non- Converters [MCI_NC], Dark blue = MCI-Converters [MCI_C]). The small, individual lines represent individual trajectories of loss of standardized Hippocampal volume (Red lines = MCI-Non-Converters [MCI_NC], Aquamarine lines = MCI-Converters [MCI_C]). Data after conversion to DAT in the MCI_C group were excluded. The x-axis was standardized in analyses but is plotted via age for ease of interpretation.

We found a significant interaction between age and cohort in predicting standardized Hippocampal volume (b = − 0.28, p < 0.001). Specifically, we found that Hippocampal volume declined more rapidly in participants who progressed to DAT than those who did not (Fig. 1).

Random effects showed variation around the intercept (*u*_*0j*_ = 0.83) and slope (*u*_*1j*_ = 0.28), and these were not correlated *r* = 0.05. The random intercept and slope model with unstructured covariance structure (AIC = 1138.8), fit better than the random intercept-only model (AIC = 1431.9), which fit better than the fixed effects model (AIC = 5943.3).

A comparison between this LME model and a fixed effects only model (simple linear regression which does not account for individual variation) highlights the significance of this better fit, since the latter finds a smaller interaction effect suggesting that it can only detect a weaker effect. (Supplementary S2.2).

**Figure 2:**
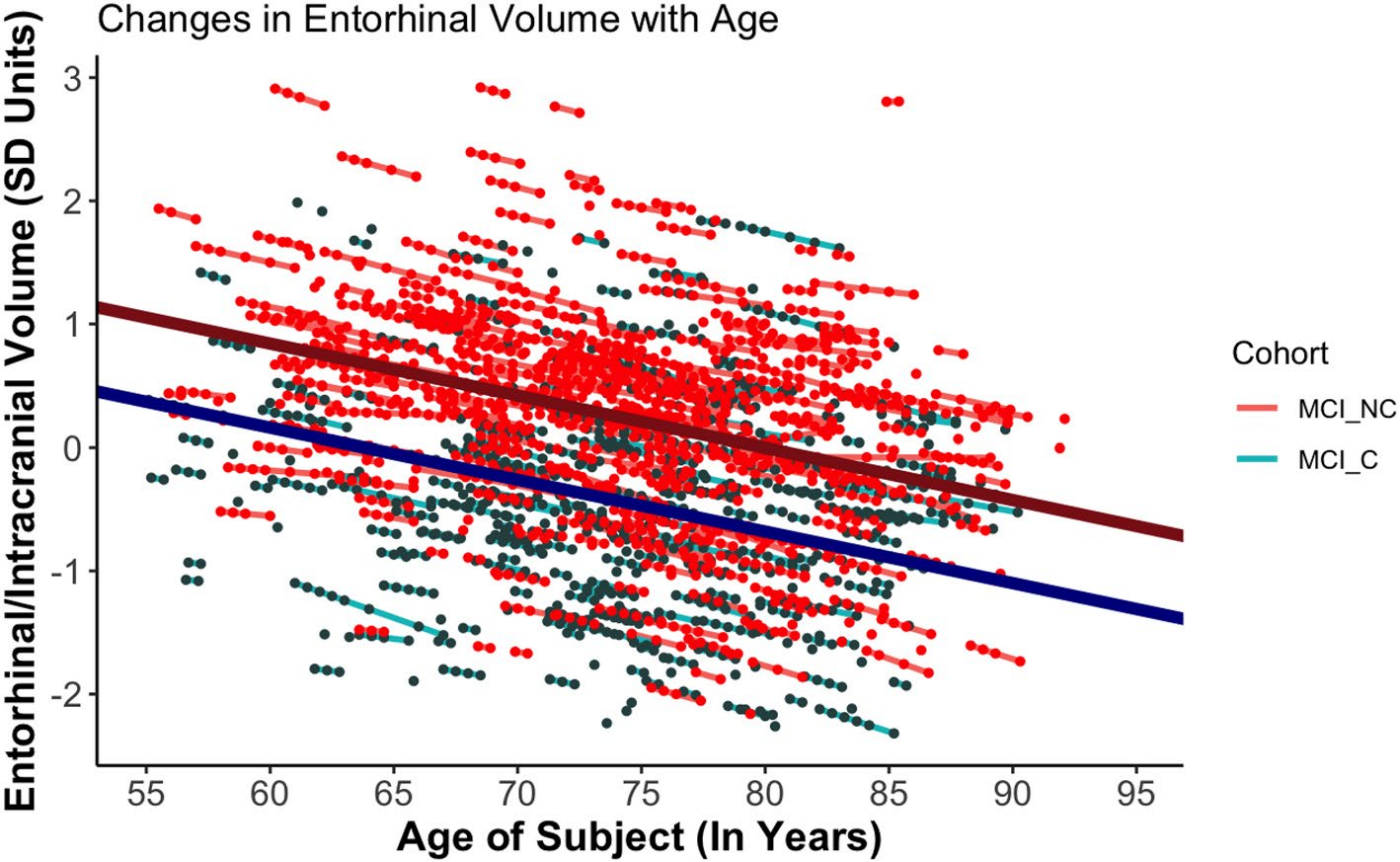
Trajectories of Decrease of EC Volume with Age. Trajectories of EC Volume Loss with Age. The trend lines represent the average trajectory of loss of standardized EC volume with age for each MCI Cohort (Dark red = MCI-Non-Converters [MCI_NC], Dark blue = MCI-Converters [MCI_C[). The small, individual lines represent individual trajectories of loss of standardized EC volume (Red lines = MCI-Non Converters [MCI_NC], Aquamarine lines = MCI-Converters [MCI_C[). Data after conversion to DAT in the MCI_C group were excluded. The x-axis was standardized in analyses but is plotted via age for ease of interpretation.

A similar LME model examining the effect of age, cohort, and their interaction on the standardized EC volume of participants with MCI did not yield a significant interaction between age and cohort (b =  − 0.002, p = 0.978). This suggests that the rates of age-related decrease of EC volume did not differ significantly in MCI-Converters and Non-Converters (Fig. 2). However, it is worth noting that Converters started at lower baseline volumes as compared to Non-Converters, suggesting that the former might have experienced a more rapid decline even before the onset of MCI in a preclinical stage.

Random effects in the EC volume model also showed variation around the intercept (*u*_*0j*_ = 0.71) and slope (*u*_*1j*_ = 0.059), and these were slightly correlated, *r* = 0.11. The random intercept and slope model with unstructured covariance structure (AIC = 3841.2), fit better than the random intercept-only model (AIC = 3845.7), which fit better than the fixed effects model (AIC = 5945.5).

Adding a fixed effect of ADNI Cohort (ADNI1, ADNI-GO, ADNI2 or ADNI3) did not have any effect on our findings. This can likely be attributed to the fact that we measure volumes as a ratio of the regional brain volume divided by the intracranial volume as observed in the same research visit. Since both volumes in the ratio are obtained based on the same scanner strength and protocol, the common elements introduced by a specific ADNI protocol may get canceled out.

We also evaluated if age of onset of MCI was related to individual estimates of rates of loss of Hippocampal and EC volumes. We found that Hippocampal volume declined more rapidly in individuals with a later age of onset (b =  − 1.87, p = 0.012). However, we found no relation between age of onset of MCI and individual rates of Entorhinal volume loss in the MCI stage.

#### Analyses of additional regions

Similar LME models as in the above analysis were fit to predict standardized Fusiform Gyrus, Occipital Lobe, Lateral Ventricles and Whole Brain volumes (Supplementary Fig. S3). Of these models, we only observed a significant interaction between age and cohort in predicting Lateral Ventricle volumes (b = 0.28, p < 0.001). This effect suggests that the enlargement of lateral ventricles with age is more rapid in patients with MCI who progress to DAT than those who do not.

Like in the previous analysis, adding a fixed effect of ADNI Cohort did not alter our findings in these models.

Additionally, we found no significant relation between the age of onset of MCI and the individual rates of change of volumes in any of the additional regions.

The fixed effects, random effects and related statistics for all 4 models are listed in Supplementary Tables S1 to S4. For all regions, adding random effects around both intercept and slope improved the model fit.

### Predicting progression to dementia of the Alzheimer’s type

#### Using rates of hippocampal and EC volume loss

Logistic regression analyses revealed that the individual rates of decrease of Hippocampal volume (M = − 0.72, SD = 0.39) significantly predicted whether or not an MCI participant would progress to DAT, *b* = − 0.47 (SE = 0.09), *p* < 0.001. For each standard deviation slow down in the rate of Hippocampal volume decline with age, the odds of the progression to dementia decreased by 37% (odds ratio = 0.63). A model that included the individual rate of loss of Hippocampal volume as a predictor (AIC = 805.79) fit better than the intercepts-only model (AIC = 830.95), *Δχ*^2^(1) = 27.17, *p* < 0.001. While the odds ratio appears large at first, this is contextualized by the fact that the SD of the individual Hippocampal slopes is also quite large—a little over half the mean itself.

**Figure 3:**
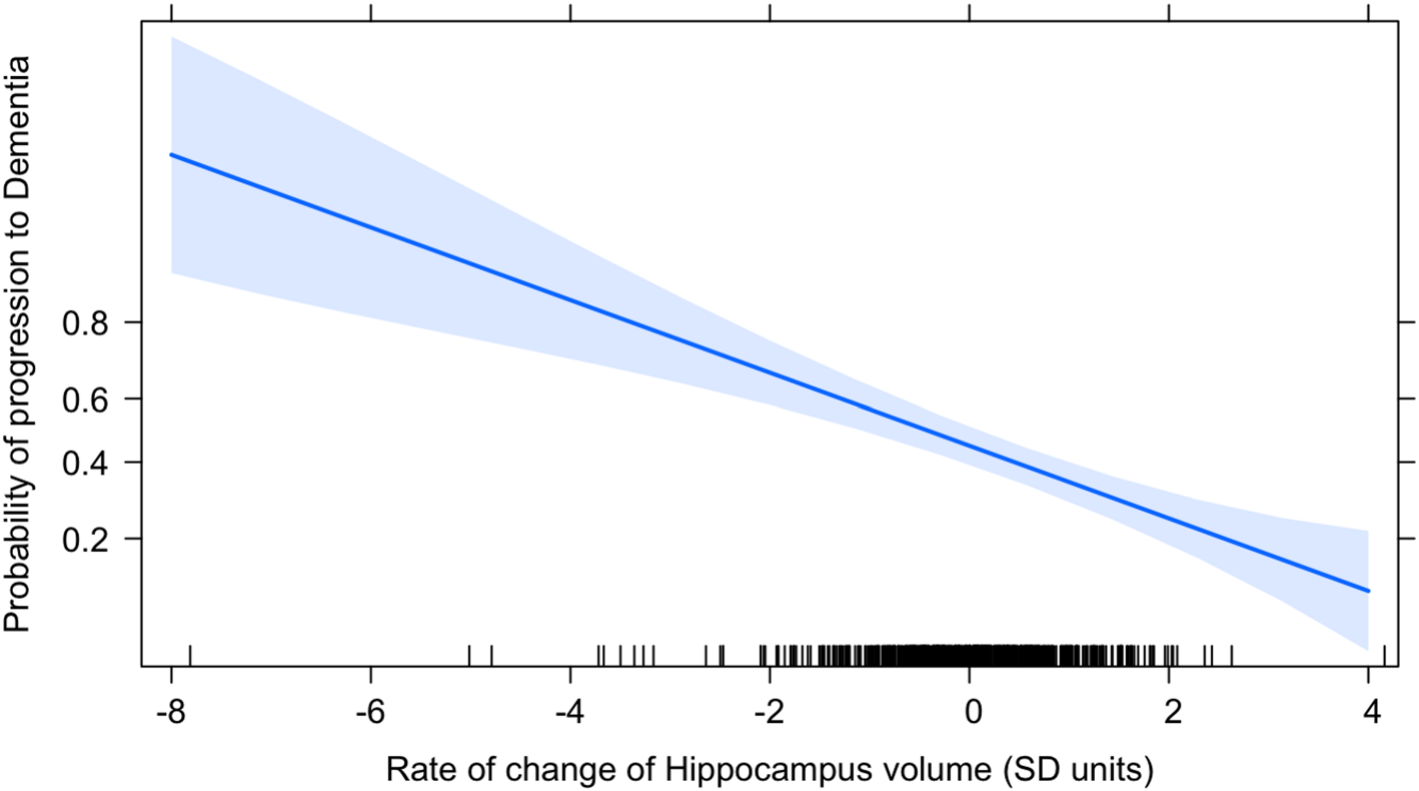
Predicting Progression to dementia—Hippocampal Rates. The trend line depicts the rate at which the probability of progression to DAT decreases as the rate of loss of Hippocampus volume becomes less negative. Since rate of change is measured in SD units, a rate of change of 0 on the x-axis corresponds to the average rate of change of Hippocampal volume across individuals (− 0.72 SD units of Hippocampal decline with each SD unit increase in age). As the rate of Hippocampal volume loss becomes more rapid than the average (negative x-axis), the probability of progression to DAT increases. As the rate of Hippocampal volume loss becomes less rapid than average (positive x-axis), the probability of progression to DAT decreases. The blue shaded area represents a pointwise 95% confidence band around fitted values.

These results suggest that individual rates of decline in Hippocampal volume with age in the MCI stage can predict progression to DAT, with the odds of progression significantly increasing as the rates become more negative (steeper) (Fig. 3).

On the other hand, individual rates of EC volume loss did not predict whether a patient with MCI would progress to DAT, *b* =  − 0.063 (SE = 0.08), *p* = 0.441. Furthermore, a model that included the individual rate of EC volume loss as a predictor (AIC = 832.36) did not fit any better than the intercept-only model (AIC = 830.95), *Δχ2*(1) = 0.60, *p* = 0.439.

#### Using rates of volume loss in additional regions

**Figure 4:**
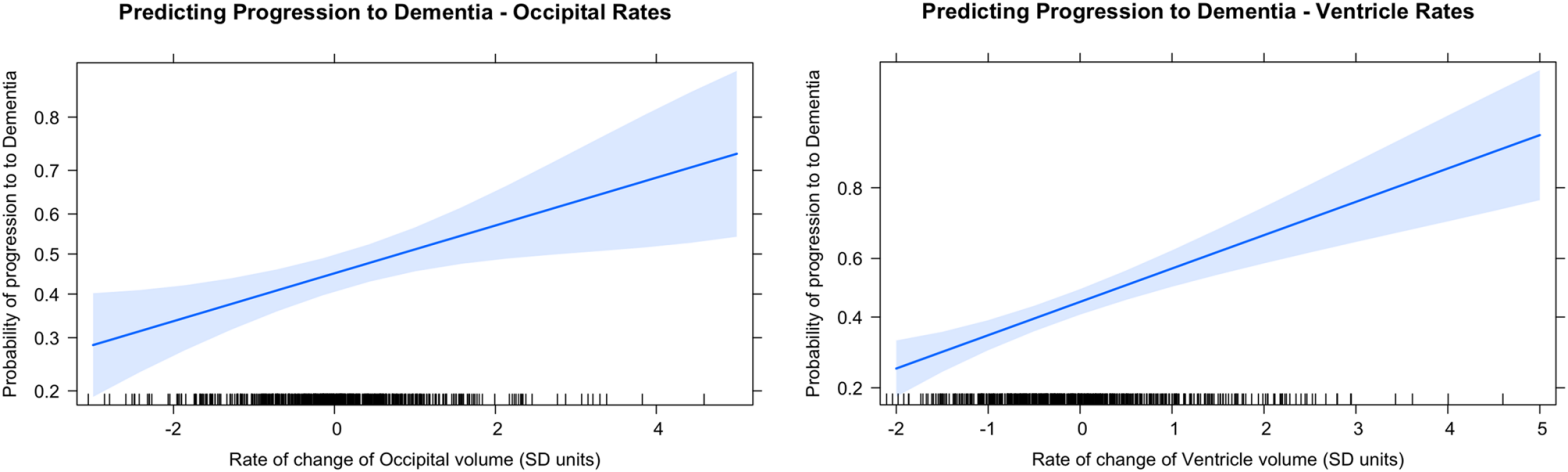
Predicting Progression to DAT using Occipital and Lateral Ventricle Volume Slopes. The trend lines depict the rate at which the probability of progression to DAT changes with the rate of change of Occipital volumes and rate of change of lateral ventricle volume (in SDs), respectively. Since rate of change is measured in SD units, a rate of change of 0 on the x-axis corresponds to the average rate of change of Occipital (left) or Ventricle (right) volumes across individuals (− 0.27 SD units of Occipital volume loss and 0.86 units of ventricle expansion with each SD unit increase in age respectively). For the Occipital rates, since the mean is *negative*, as the rate of Occipital *volume loss* becomes more rapid than the average (negative x-axis), the probability of progression to DAT decreases, and it becomes becomes less rapid than average (positive x-axis), the probability of progression to DAT increases. For the Ventricle rates, since the mean is *positive*, as the rate of *ventricle expansion* becomes more rapid than average (positive x-axis), the probability of progression to DAT increases. As the rates of expansion become less rapid (negative x-axis), the probability of progression decreases. The blue shaded areas represent a 95% pointwise confidence band around fitted values.

Lateral Ventricle slopes (M = 0.86, SD = 0.47) significantly predicted progression to DAT, *b* = 0.46 (SE = 0.09), *p* < 0.001 (Fig. 4). For each standard deviation increase in the rate of volume gain, the odds of progression to dementia increased by 59% (odds ratio = 1.59). A model that included the individual rate of lateral ventricle volume gain as a predictor (AIC = 804.02) fit better than the intercepts-only model (AIC = 830.95), *Δχ*^2^(1) = 28.93, *p* < 0.001. Note that our study defines lateral ventricle slopes as the change in the ratio of lateral ventricle volume to the ICV (M = 0.03, SD = 0.01) in standard deviations, for each standard deviation increase in age (M = 75.4, SD = 7.6 years).

Occipital slopes (M =  − 0.27, SD = 0.18) also significantly predicted progression to DAT, but in a direction opposite to that seen in the Hippocampus and EC, *b* = 0.24 (0.08), *p* < 0.01 (Fig. 4). For each standard deviation unit increase in the rate of loss, the odds of the progression to dementia *decreased* by 27% (odds ratio = 1.27). A model that included the individual rate of occipital volume loss as a predictor (AIC = 824.47) fit better than the intercepts-only model (AIC = 830.95), *Δχ*^2^(1) = 8.48, *p* < 0.01. Note that our study defines occipital slopes as the change in the ratio of occipital volume to the ICV (M = 0.01, SD = 0.002) in standard deviations, for each standard deviation increase in age.

On the other hand, neither Fusiform Gyrus slopes (*b* = − 0.04 [0.08], *p* = 0.587) nor whole brain slopes (*b* =  − 0.16 [0.08], *p* = 0.059) significantly predicted progression to DAT.

### Evaluating the link between rates of structural and cognitive decline

#### Mapping trajectories of cognitive decline

**Figure 5:**
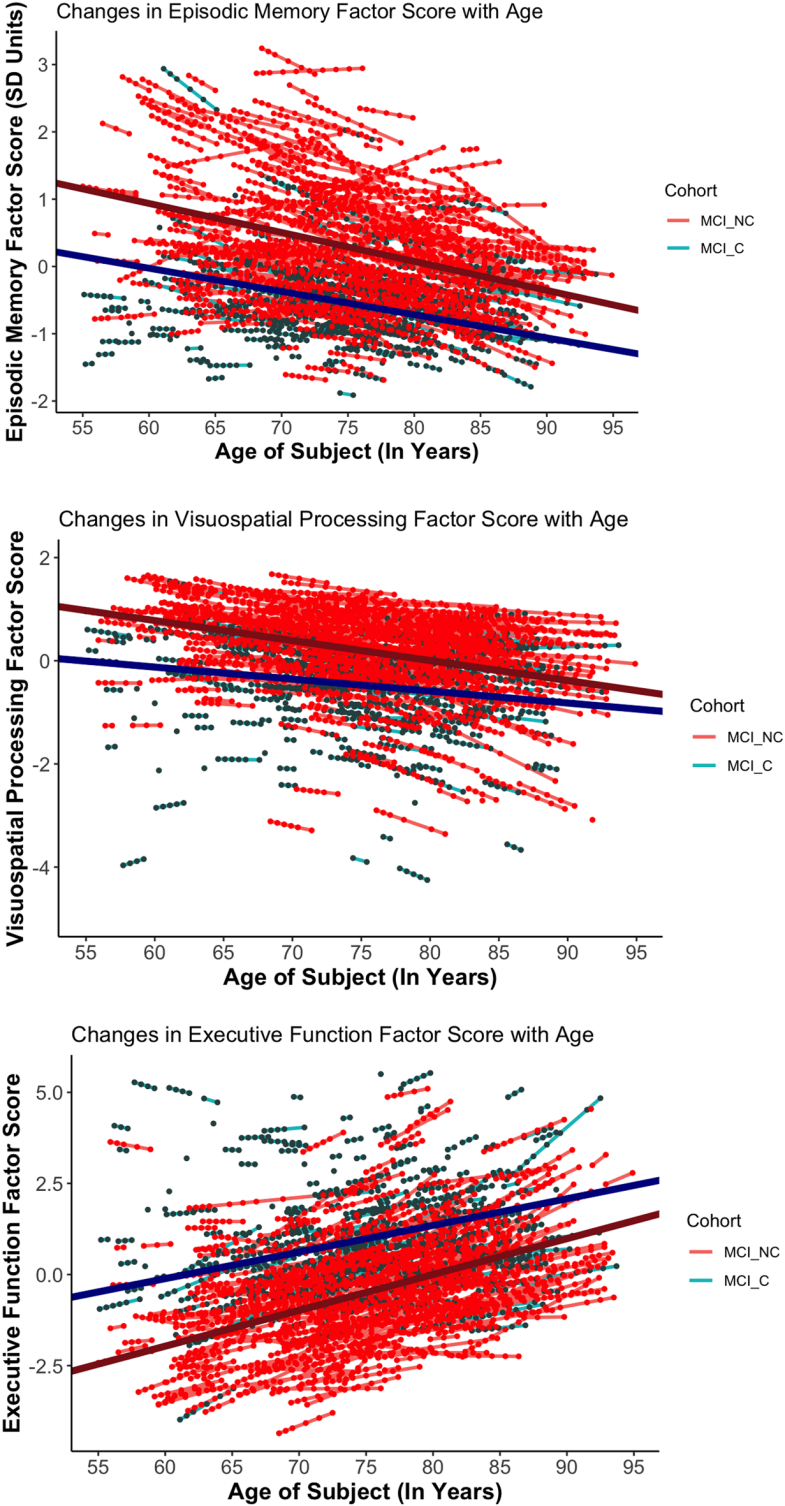
[Top to Bottom] Trajectories of Episodic Memory, Visuospatial Functioning, and Executive Function Decline with Age. For Executive Function scores, a higher score implies poorer cognition. The trend lines represent the average trajectory of change in cognitive factor scores with age for each MCI Cohort (Dark red = MCI-Non Converters [MCI_NC], Dark blue = MCI-Converters [MCI_C]). The small, individual lines represent individual trajectories of change of cognitive factor scores (red lines = MCI-Non Converters [MCI_NC], Aquamarine lines = MCI-Converters [MCI_C]). The x-axis was standardized in analyses but is plotted via age for ease of interpretation.

LME models were used to examine the effect of age, cohort, and their interaction on each of the 3 identified cognitive factor scores—episodic memory, visuospatial processing, and executive function—in patients with MCI (Fig. 5).

We found a significant decline in cognitive performance with age across all 3 cognitive factors, with episodic memory factor scores (b = − 0.32, p < 0.001), visuospatial processing scores (b =  − 0.29, p < 0.001), and executive function factor scores (b = 0.74, p < 0.001) all declining with age.

The executive function factor comprises exclusively trail making scores which load positively onto it. Since trail-making tasks score participants on the time they take to complete a task, a more positive executive function factor score indicates worse cognitive performance. For the other two cognitive factors, a higher score indicates better cognitive performance.

Additionally, we found that on average, patients who progressed to DAT exhibited poorer performance than those who did not across all 3 factors, with MCI-Converters having poorer episodic memory factor scores (b = − 0.83, p < 0.001), visuospatial processing factor scores (b =  − 0.66, p = p < 0.001) and executive function factor scores (b = 1.46, p < 0.001) than MCI Non-Converters.

However, we did not find a significant interaction between age and cohort in predicting any of these 3 cognitive factors—episodic memory factor scores (b = 0.06, p = 0.296), visuospatial processing factor scores (b = 0.12, p = 0.136) and executive function factor scores (b = − 0.19, p = 0.101). This suggests that the rates of decrease in cognitive performance with age in the MCI stage did not depend on whether the patient progressed to DAT or not.

However, it is worth noting that MCI-Converters begin with lower baseline scores across all 3 cognitive measures, suggesting the possibility that Converters might have undergone a more rapid decline prior to the onset of MCI, in a preclinical stage, in a pattern similar to volume loss in the EC.

We also evaluated if age of onset of MCI was related to individual estimates of rates of decline in cognitive performance on all identified cognitive factors. We found that episodic memory declined more rapidly in individuals with a later age of onset (b = − 5.06, p < 0.001). We also found that executive function performance decreased more rapidly in individuals with a later age of onset (b = 3.07, p < 0.001). However, we found no relation between age of onset of MCI and individual rates of visuospatial processing decline in the MCI stage.

#### Correlations between rates of structural and cognitive decline

##### Correlations with hippocampal and EC rates of decline

**Figure 6:**
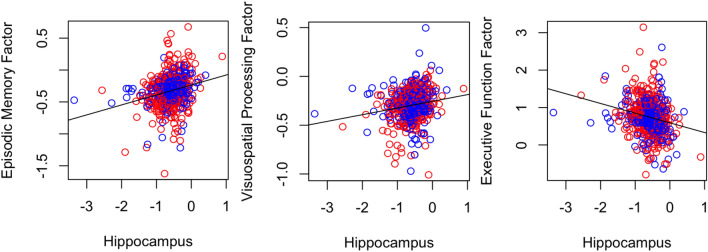
Correlations between individual rates of Hippocampal Volume Loss and Cognitive Decline. MCI-Converters in blue, and MCI-Non Converters in red. Each dot represents an individual.

Individual rates of Hippocampal volume loss were significantly correlated with the individual rates of decline in all 3 cognitive factors, in which a more rapid loss of Hippocampal volume was correlated with a more rapid decline in episodic memory (r = 0.24, p < 0.001), visuospatial functioning (r = 0.17, p < 0.001) and executive function (r =  − 0.21, p < 0.001) (Fig. 6). Note that a Higher Executive Function factor score corresponds to worse cognitive performance.

**Figure 7:**
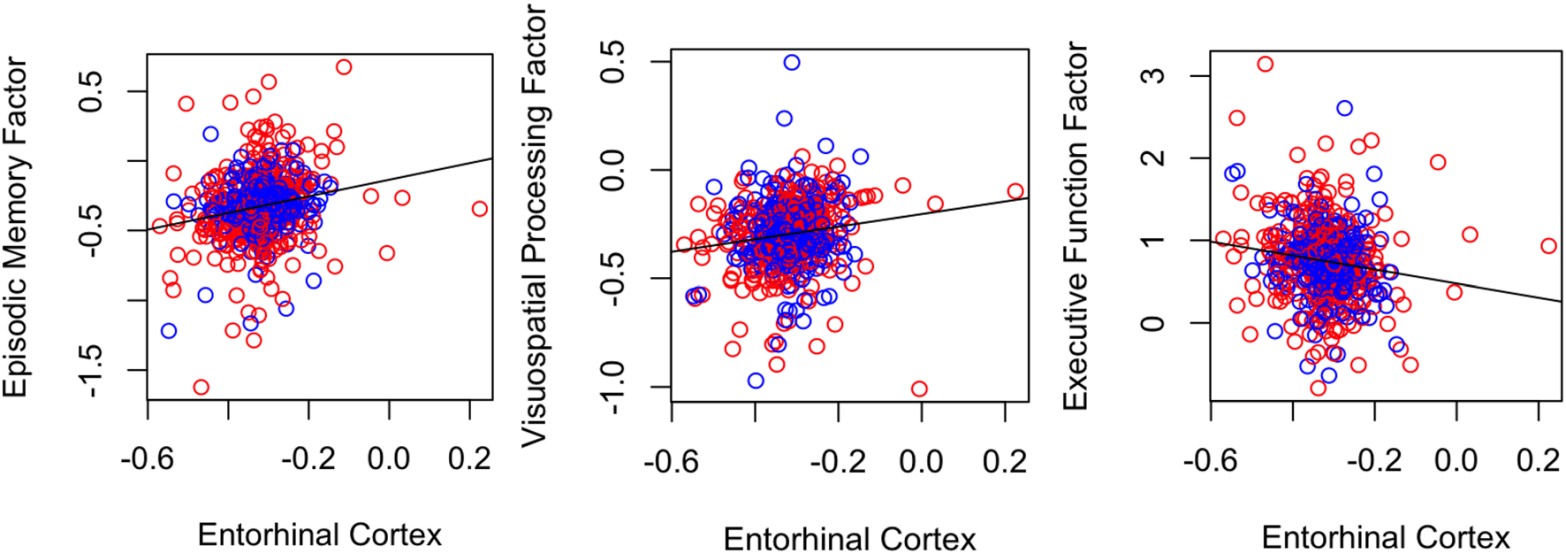
Correlations between individual rates of Entorhinal Volume Loss and Cognitive Decline. MCI-Converters in blue, and MCI-Non Converters in red. Each dot represents an individual.

A similar trend was seen for individual rates of EC volume loss, in which a more rapid loss volume was correlated with a more rapid decline in episodic memory (r = 0.19, p < 0.001), visuospatial functioning (r = 0.14, p < 0.001) and executive function (r =  − 0.15, p < 0.001) (Fig. 7).

##### Correlations with additional region rates of decline

The individual estimates of structural decline of many of the additional regions were also significantly correlated with rates of cognitive decline across all 3 factors, with more rapid cognitive decline accompanying more rapid structural decline. More rapid rates of Fusiform Gyrus volume loss were correlated with more rapid rates of decline of episodic memory (r = 0.12, p < 0.01), visuospatial functioning (r = 0.19, p < 0.001), and executive function (r = − 0.16, p < 0.001). More rapid rates of volume increase of the ventricles was correlated with more rapid rates of decline of visuospatial functioning (r = − 0.14, p < 0.001), and executive function (r = 0.16, p < 0.001) but not with rates of decline of episodic memory (r = − 0.03, p = 0.38). More rapid rates of loss of whole brain volume were correlated with more rapid rates of decline of episodic memory (r = 0.12, p < 0.01), visuospatial functioning (r = 0.16, p < 0.001), and executive function (r = − 0.18, p < 0.001).

The major exception was that rates of volume loss in the Occipital Lobe were not significantly correlated with rates of change of episodic memory (r = 0.04, p = 0.31), visuospatial functioning (r < 0.01, p = 0.977), or executive function (r = − 0.07, p = 0.087).

## Discussion

### Comparing rates of regional volume loss in MCI-Converters and Non-Converters

The rate of Hippocampal volume loss with age is significantly more rapid in MCI Converters than in Non-Converters, suggesting its potential as a biomarker for disease progression. The fact that this finding did not rely on post-conversion data from MCI-Converters emphasizes the distinction in Hippocampal volume change rates during the MCI stage itself. In contrast to the Hippocampus, we found no significant difference in the rate of change of EC volume between converters and non-converters. Given that the neuropathological changes in AD tend to originate in the EC^7,35^, this finding might imply that differences in EC rates of volume loss are more evident at younger ages preceding MCI onset. Recent studies have identified specific subfields in the EC affected by tau pathology in the preclinical stage—a stage that precedes MCI during which disease-related neuropathology has begun but clinical symptoms are yet to manifest^36^. There is also evidence that entorhinal dysfunction, that precedes neurodegeneration, begins in adults as young as 18–30 years^37^. Research quantifying the times at which disease related atrophy, distinct from age related atrophy, begins in the EC suggests that it begins up to 8–11 years prior to MCI onset, much earlier than in the Hippocampus which begins only 2–4 years earlier^38,39^. This age period is not represented in our sample, which was selected to focus on MCI, but this is a clear hypothesis that can be tested in future work. An alternative explanation for the lack of an interaction effect in predicting EC volumes could be the truncation of followup visits in MCI-Converters (post conversion to DAT) and not in MCI-Non Converters. However, we found no significant interaction effect even after accounting for this difference (Supplementary Sect. S2.1).

We also found that the lateral ventricles enlarge at a more rapid rate in converters than in non-converters. This is consistent with existing research that proposes ventricle volumes as a potential biomarker for disease progression with individuals with MCI demonstrating intermediate lateral ventricle volumes as compared to those with normal cognition or DAT^31–33^. It has been posited that this might be because ventricle enlargement acts as a compensatory mechanism wherein the ventricles act as a sink for faulty CSF with Aβ deposits before being cleared out^32,40^.

Additionally, across all evaluated regional volumes, the individual differences in baseline volumes and rates of volume change were notable. Some MCI-Non Converters experienced a more rapid loss of regional brain volume than the average MCI-Converter. This suggests the likely influence of cognitive reserve and other factors in mitigating cognitive decline despite structural decline, highlighting the need for further research into these underlying factors.

### Rates of regional volume loss as potential biomarkers for progression to DAT

Individual rates of Hippocampal volume loss significantly predict progression to DAT, whereas Entorhinal rates of volume loss do not. The inability of rates of Entorhinal volume loss to predict conversion might be attributed to the early onset and stabilization of these changes before the MCI stage as discussed above. We also found that rates of volume increase in the lateral ventricles were able to predict progression to DAT. One surprising result was that more rapid rates of Occipital Lobe volume loss significantly *reduced* the risk of progression to DAT in the MCI stage. Given the unexpected direction of these findings, we probed them further by separately evaluating if unstandardized Occipital volume rates and ICV rates could predict progression to DAT, instead of their ratio. Interestingly, we found that neither rate alone could significantly predict progression. Additionally, we wondered whether the less rapid rate of Occipital volume in MCI-Converters could be explained as a compensatory mechanism compensating for the more rapid decline in other regions like the Hippocampus or possibly the EC. However, we did not find a negative correlation between the individual rates of decline of the Occipital Lobe and the Hippocampus, or the EC (using both standardized and unstandardized individual rates of decline). We are unsure what caused these findings and these should be explored in future research.

### Linking rates of structural and cognitive decline

We found significant correlations between individual rates of structural and cognitive decline in MCI patients. The strongest correlations were observed in regions crucial to the related cognitive function, like the hippocampus and EC with episodic memory. Likewise, volume loss in the fusiform gyrus and hippocampus was more strongly associated with a decline in visuospatial processing than volume loss in other regions. However, cognitive decline was also related to volume changes in regions not typically associated with these functions. These relationships align with findings in healthy aging, which show that age-related variation in cognitive performance is shared among various brain markers^41^.

Interestingly, the cognitive decline—in episodic memory, visuospatial processing, and executive function—occurred at similar rates in MCI-Converters and Non-Converters. This could indicate that the more accelerated decline in cognition among converters occurred before the first testing session, possibly during the preclinical stage. The fact that MCI-Converters begin at a lower level of cognition supports this hypothesis. This hypothesis also aligns with studies showing the onset of cognitive decline 3 to 7 years before an MCI diagnosis^42^ and mirrors the trends observed in EC volume loss.

### Limitations

This study’s primary limitation is its focus on neural and behavioral measures that we obtained after the MCI diagnosis, neglecting changes during preclinical stages. We speculate that both EC volume loss and cognitive decline might be more pronounced in Converters than Non-Converters pre-diagnosis. Future research in large longitudinal data sets could retrospectively examine these aspects in MCI patients before their MCI diagnosis.

Another limitation is the study’s exclusive focus on neurodegeneration as a biomarker for progression to DAT during the MCI stage, without incorporating neuropathological changes related to Amyloid-β or Tau proteins. This aspect is crucial, as understanding the relationship between these neuropathological markers^43^ and the neurodegeneration trajectories identified in MCI patients could significantly enhance mechanistic theories of disease progression. The inclusion of these biomarkers could provide a more nuanced understanding of the pathophysiology underlying MCI and its progression to DAT.

This study also does not evaluate sub-field specific atrophy in the Hippocampus. Recent work suggests that the rate of atrophy of specific Hippocampal subfields might be a better biomarker for progression to DAT in the MCI stage than that of the whole Hippocampus^44–46^. Specifically, MCI-Converters seem to be categorized by a selective atrophy of the subiculum and tail regions as compared to Non-Converters^45^. Volumes of certain other subfields including the parasubiculum, CA2/3 and the fimbria do not seem to distinguish between MCI-Converters and Non-Converters^44^. Exploring the atrophy of specific hippocampal subfields within our longitudinal LME model framework is an important next step.

Furthermore, the analysis relied on relatively broad neural and behavioral measures. More granular future studies could, for example, differentiate volume loss in specific hippocampal subfields and entorhinal cortex areas, which are variably affected by disease progression^11,47, 48^. Similarly, expanding the range of cognitive tasks in future research could elucidate the relationship between specific cognitive declines and regional volume loss.

### Conclusion

In this study, we examined rates of volume loss in specific brain regions in MCI patients who converted to DAT and in MCI patients who did not. We adopted a longitudinal approach in which we examined within-participant change over a few years, we utilized a large sample of MCI patients (both converters, and non-converters), and we fit linear mixed effects models to incorporate and account for individual differences in initial volume and in rate of volume loss.

We demonstrated that longitudinal volume loss in the hippocampus during the MCI stage distinguishes and predicts patients who convert to DAT from patients who do not, whereas volume loss in the entorhinal cortex and numerous additional regions does not. Furthermore, individual differences in volume loss are significantly associated with individual differences in cognitive decline. We also demonstrated the importance of incorporating individual differences, both in initial volume and in rate of volume loss, to accurately model these brain and behavioral processes.

## Methods

### Participants

Our study analyzes data from the Alzheimer’s Disease Neuroimaging Initiative (ADNI). The data from ADNI are available upon registration and compliance with the data usage agreement (adni.loni.usc.edu). All ADNI studies are conducted according to the Good Clinical Practice guidelines, the Declaration of Helsinki, and U.S. 21 CFR Part 50 (Protection of Human Subjects), and Part 56 (Institutional Review Boards). Written informed consent was obtained from all participants before protocol-specific procedures were performed. The ADNI protocol was approved by the Institutional Review Boards of all participating institutions. The investigators within the ADNI contributed to the design and implementation of ADNI and/or provided data but did not participate in analysis or writing of this report. A complete listing of ADNI investigators and affiliations can be found at http://adni.loni.usc.edu/wp-content/uploads/how_to_apply/ADNI_Acknowledgement_List.pdf. Our analysis was approved by the ADNI Data Sharing and Publications Committee.

We analyzed data from 758 MCI participants from the ADNI database (adni.loni.usc.edu). We used data from across 4 available cohorts of the ADNI Database—ADNI1, ADNI-GO, ADNI2 and ADNI3—listed here in the sequence they occurred in. Each cohort involved slightly different clinical visit frequencies, and study and MRI scanner protocols (https://adni.loni.usc.edu/methods/documents/). For example, ADNI 1 involved 1.5T MRI scans, with 25% of its participants additionally being scanned with a 3T scanner. ADNI-GO, ADNI-2 and ADNI-3 all involved 3T scans with slightly different acquisition protocols. (https://adni.loni.usc.edu/methods/mri-tool/mri-analysis/). Each new cohort included a new set of participants, and a set of individuals who rolled over from the previous cohort.

ADNI uses the following criteria to assign an MCI diagnosis to a participant at an assessment visit: Either the participant or the study partner reported memory complaints; the participant had an abnormal memory function score on the Wechsler Memory Scale (adjusted for education); the participant had an Mini Mental State Examination (MMSE) score between 24 and 30; and the participant had a global Clinical dementia Rating (CDR) of 0.5 (https://clinicaltrials.gov/ct2/show/NCT00106899). Four participants who were assigned an MCI diagnosis despite not meeting these criteria were excluded from this study.

We further divided this group of MCI participants into 2 cohorts—MCI-Non Converters (n = 400 [39.5% female], age of MCI onset in years [M = 73.00, SD = 7.38], years of education [M = 16.04, SD = 2.82], race [94.7% White, 3% Black,1.8% Asian, 0.5% Mixed Race]) and MCI-Converters (n = 358 [41.9% female], age in years [M = 73.99, SD = 6.80], years of education [M = 15.89, SD = 2.77], race [95% White, 3% Black, 1.7% Asian, 0.03% Mixed Race]). We defined MCI-Non Converters (MCI-NC) as participants who retained their MCI diagnosis for all follow-up visits after first attaining it. We only included participants who had at least 2 years of follow-ups after their first MCI diagnosis ^49–51^. We defined MCI-Converters (MCI-C) as participants who transitioned from an MCI to a DAT diagnosis at some point and then retained their DAT diagnosis until the last known followup visit.

We used the Dx_bl (Baseline Diagnosis) and Diagnosis (Diagnosis at current research visit) variables from ADNI’s ADNIMERGE datasheet to identify MCI-Converters and Non-Converters. We did this by writing a script that traversed through the diagnoses assigned to each individual at each subsequent visit, and using a counter Dx_Change to measure how many times the individual’s diagnosis changed in their duration through the study. The script uses each individual’s baseline diagnosis, the count of the number of times their diagnosis changes and their final diagnosis (Dx_final) to assign them to a category. For example, MCI-Non Converters included individuals who either had a Dx_bl = “MCI” and a Dx_change = 0, or a Dx_bl = “CN”, Dx_change = 1, and Dx_final = MCI.

All MCI participants engaged in multiple research visits over the period they were enrolled in the study. These research visits could include clinical testing or an MRI scan or both. The frequency of repeat visits differed based on the ADNI cohort the participant belonged to. Repeat visits are referenced relative to the baseline visit. Note that our study does not make use of data from the screening visit to assign a participant to a diagnostic category. ADNI1 involved clinical and scan visits at the baseline visit, 6 month and 12 month visits, and once a year thereafter up to 48 months. ADNI-Go and ADNI2 involved clinical visits at the baseline, 6 and 12 month visits and annually thereafter, while the scans occurred at a 3 month visit, 12 month visit and annually thereafter as long as funding allowed. In ADNI3 both clinical and scan visits occurred annually starting from the baseline visit. Additionally, the data of participants included in this study spanned across multiple ADNI cohorts longitudinally.

On average, the MCI-Non Converters (MCI-NC) had an average of 6.03 (SD = 2.65) research visits per participant, with an average period of 10.27 months between subsequent research visits and a followup period of up to 180 months spanning across cohorts. The MCI-Converters (MCI-C) had an average of 6.44 (SD = 2.5) research visits per participant, with an average period of 9.23 months between subsequent research visits and a followup duration of up to 168 months. After removing post-conversion visits, MCI-Converters had an average of 3.52 (SD = 2.1) visits per participant, with an average period of 7.37 months between visits and a maximum follow up duration of 144 months.

Finally, we used the baseline visits of 534 CN ADNI participants to identify latent cognitive constructs, in order to evaluate the trajectories of the decline of these constructs for participants in the MCI stage (Research Question C). We included participants who consistently retained a ‘CN’ diagnosis throughout all their research visits, only retaining participants who had at least 2 years of follow-ups after their first research visit.

### Neuroimaging measures

Hippocampal, EC, Occipital Lobe, Fusiform Gyrus, Whole Brain, and Lateral Ventricles volume measures were extracted from the ADNI Database. ADNI acquired these measures by using Freesurfer cortical parcellation on structural MRI scans from each research visit (Supplementary Methods). We standardized all regional volumes by dividing them by intracranial volume (ICV). We then z-scored (mean-centered and divided by the standard deviation) these ratios across the whole sample. These ratios are hereafter referred to as *standardized volumes*.

### Neuropsychological measures

We performed a factor analysis on 13 item-wise cognitive test scores from the ADNI Neuropsychological Battery to perform a factor analysis (Score Selection—Supplementary Methods). We included two measures of visuospatial processing from the clock-drawing test—the clock-drawing command score and the clock-drawing copy score^52^. We also included two measures of executive function and processing speed from the trail-making task—trail-making test parts A and B^53^**.** Finally, we included 9 measures of episodic memory from the Rey Auditory and Verbal Learning Test (RAVLT)^54^. These measures of progressive recall and recognition are described in detail in the Supplementary materials.

For items from both the RAVLT and Clock Drawing tasks, a higher score indicates better cognitive performance. However, for items from the Trail-Making test, scores depict the time taken to complete the task. Consequently, a higher score indicates poorer cognitive performance.

### Linear mixed effects (LME) models

Linear Mixed Effects (LME) models are extensions of simple linear models, which account for both fixed effects and random effects, that adjust for dependencies due to multiple time-linked assessments coming from the same participant. The fixed effects capture the dependency-adjusted average effect of each predictor on the outcome, and the random effects capture variance in the effects (in this application, around intercepts and slopes). In the growth curve models applied here, which are a special case of LME models, this variance reflects differences across individuals. Specifically, in our models, random effects capture individual deviations of baseline volumes from the mean volume, and in rates of volumetric change from the mean change (slope). Empirical Bayes estimation was then used to generate priors that are updated using Bayes theorem based on an individual participant’s data in order to estimate individuals’ intercept and slope values^55^. The resulting estimates are typically significantly better than would be obtained using only the participant’s own data (which typically only includes a few data points). We used the lmer() function from the lme4 package in R to fit the LME models and estimate individual values^56^.

The use of LME models over a more conventional longitudinal analysis approach like a repeated measures ANOVA presents several advantages. Firstly, it can accommodate common issues seen in real-world longitudinal data^57^. These models can handle participants joining the study at different points and having variable durations between subsequent research visits in participants, as seen in different protocols corresponding to different ADNI cohorts. They can also accommodate missing or incomplete data within the participants’ followup periods. They also allow for accommodating participants who have different lengths of followup periods which allows us to include newer and more long-term ADNI participants within the same analyses. With these accommodations, these models allow for the incorporation of a larger amount of data that would otherwise be left out in more constrained methods, which in turn allows for larger power in the analysis and more replicable, generalizable effects.

Secondly, LME models allows for the Bayes Estimation of individual baseline and rate of change estimates (random effects) as a relative measure in comparison to the group average (fixed effects)^58^. These estimates not only provide a reference for how much each individual deviates from the group averaged effects, but also how much individual variance is present in the effect being studied in the group. In groups with a lot of inter-individual variance, an effect that does not account for this variance might not be valid or reliable. Not all individuals with MCI present with the same degrees of cognitive or structural decline, and also demonstrate differences in other demographic factors. LME models allow us to see if there is still an effect after accounting for these individual dependencies in the data, making their effects more robust than models that do not.

The use of LME models in our study allows us to measure what the MCI stage looks like at the individual level by estimating how the structure of the brain, and cognitive performance change over time within the individual. To ensure that the comparison is at the individual level, we use the structural or cognitive measures corresponding to each individual’s first visit included in the study as their own baseline, such that the rates of change for each individual correspond to how much they deviate from their own baseline over their followup visits. This is particularly useful since our model accommodates individuals starting at different points of time, with different regional brain volumes and different levels of cognitive decline wherein a single group-averaged baseline metric might not serve as a good reference point. However, in visualizing group-averaged baselines, the intercept corresponding to each MCI cohort in the growth curve plots can be interpreted to signify the baseline value for each group.

Finally, it is important to note that the ability of the LME approach to estimate individual trajectories (i.e., slopes and intercepts) of change in volume or cognitive ability over time, also allows for the use of these individual estimates in subsequent analyses. For example, we are able to use these individual estimates to carry out the analyses described in “Using logistic regression to predict progression to dementia from individual rates of hippocampal and EC volume loss” and “Relationship between rates of hippocampal and EC volume loss and rates of cognitive decline” sections (using individual rates of change in regional brain volume to predict progression to DAT in the MCI stage, and evaluating the link between individual rates of change in brain structure and cognition).

### Mapping trajectories of age-related brain volume loss in MCI-converters and non-converters

To address *Research Question A*, we fit growth curve models to regional volume data of MCI participants to map both the average trajectories (i.e., fixed effects) of decline for each group—MCI-C and MCI-NC—and to obtain estimates of individual trajectories of decline across MCI participants. We fit separate LME models for volumes in each of the 6 regions of interest—Hippocampus, EC, Occipital Lobe, Fusiform Gyrus, Lateral Ventricles, and the Whole Brain. These models were fit to predict standardized regional volumes (regional volume divided by intracranial volume, and then z-scored across the whole sample) using the participant’s age, cohort (0 = MCI-NC, 1 = MCI-C), and their interaction. Age was also z-scored in this analysis. We only used pre-conversion study visits for patients in the MCI-C group to limit the analysis to the MCI stage.

We also included ADNI Cohort as a fixed effect variable that could take on one of 4 values (ADNI1, ADNI-GO, ADNI2 and ADNI3) to check for any differences in outcomes due to scanner strength or protocols.

We were particularly interested in two overarching effects. First, we tested whether there was an overall difference between MCI-C and MCI-NC participants in the rates of volumetric change in each of these regions (i.e., fixed effects). A significant interaction between age and cohort implies that the rate of change of regional volume with age depends on their MCI cohort, and consequently on whether they progress to DAT in the future. Second, we evaluated whether the incorporation of individual differences (i.e., estimation of random effects) would improve the model’s fit to the data, according to the Akaike information criterion (AIC).

Additionally, we sought to evaluate if the age of onset of MCI affected the rates of change of selected brain regions over time. For this, we regressed individual estimates of the rates of change of volumes onto the age of onset of MCI. For individuals with a baseline diagnosis of CN, the age of onset was set as the age at their first visit with an MCI diagnosis. For individuals with a baseline diagnosis of MCI, their age of onset was set as their age at their first visit in the study.

### Using logistic regression to predict progression to dementia from individual rates of hippocampal and EC volume loss

To address *Research Question B*, we tested whether estimates of individual rates of volume loss in any of the regions of interest predicted progression to dementia in the MCI stage. For this, we first fit separate LME models predicting standardized regional volumes using only the individual’s age and then used the individual estimates of the rate of change as predictors of the MCI cohort in a logistic regression model. We fit separate logistic regression models for each region of interest. These regional rates of change of volume were used as predictors in separate models because some of them were correlated with each other. For these models, we used the likelihood ratio test to determine the significance of predictors entered in subsequent models and then estimated effect sizes with odds ratios.

The LME models in this analysis differ from the ones described in “Mapping trajectories of age-related brain volume loss in MCI-converters and non-converters” section because they do not include cohort-based predictors. This was done to avoid any circularity in predictions.

### Relationship between rates of hippocampal and EC volume loss and rates of cognitive decline

To address *Research Question C,* we began by conducting a principal axis factor analysis with orthogonal (varimax) rotation on the 13 item-wise scores from 594 CN participants from the ADNI Neuropsychological Battery. Based on an evaluation of the correlation matrix and a scree plot (Supplementary Fig. S1), 3 factors were extracted: one based on the auditory and verbal learning tasks which we refer to as the *episodic memory factor*, one based on the clock tasks which we refer to as the *visuospatial processing factor*, and one based on the Trails making tasks which we refer to as the *executive function factor* (Table 1). Together, these factors explained 57% of the variance, 39% by the episodic memory factor, and 9% each by the visuospatial processing and executive function factors.

**Table 1:** Factor loadings.

Item	Episodic memory	Visuospatial processing	Executive function
AVTOT3	0.88		
AVTOT4	0.88		
AVTOT5	0.82		
AVTOT6	0.82		
AVTOT2	0.79		
AVDEL30MIN	0.75		
AVTOT1	0.6		
AVTOTB	0.57		
AVDELTOT	0.49		
COPYSCOR		0.99	
CLOCKSCOR		0.36	
TRABSCOR			0.77
TRAASCOR			0.62

Loadings are thresholded at 0.3. The AVTOT 1 to 5 scores indicate performance on 5 sequential trials of recalling items from a list (list A) of 15 items that is read out to participants. AVTOTB indicates recall performance on a new list (list B) of 15 items, and AVTOT6 indicates recall of list A items after list B items are introduced. AVDEL30MIN indicates list A recall after 30 min following AVTOT6, and AVDELTOT indicates performance on recognition of list A words in a random list of words. CLOCKSCOR and COPYSCOR represent performance on drawing a clock that displays a given time, and copying a drawing of a given clock respectively. TRAASCOR and TRABSCOR record the time taken to sequentially join a set of circles that go from the numbers 1–25, and an alternating pattern of the numbers 1 to 13 and letters A to L respectively.

We then extracted factor scores for each study visit of each MCI participant for each of the 3 orthogonal factors. Factor scores were computed as the factor-loading weighted sum of the standardized cognitive scores (mean-centered and divided by standard deviation)^59^.

To evaluate how the latent cognitive constructs associated with the identified factors declined over time, we fit separate LME models for each factor with age, cohort, and their interaction predicting factor scores across repeated visits of each participant in the MCI stage. Finally, to evaluate the link between individual rates of structural and cognitive decline, we examined zero-order correlations between the two. More specifically, we looked at all pairs of correlations between individual rates of volumetric change of the 6 regions of interest and the individual rates of decline of all 3 cognitive factor scores.

We also sought to evaluate if the age of onset of MCI affected the rates of cognitive decline over time. For this, we regressed individual estimates of the rates of decline of cognitive performance on all 3 identified factors onto the age of onset of MCI separately.

### Supplementary Information

Supplementary Information.

## Data Availability

The Data used in this study was obtained from the Alzheimer’s disease Neuroimaging Initiative (ADNI) with appropriate permissions. The data are available from the ADNI database (adni.loni.usc.edu) upon registration and compliance with the data usage agreement. All ADNI data are shared without embargo through the LONI Image and Data Archive (IDA), a secure research data repository. For up-to-date information, see https://adni.loni.usc.edu/.

## Code availability

The code used in this project will be made available on request. Please reach out to the corresponding author at shreyara@umich.edu.
