# Supplementary material for: Estimating individual trajectories of structural and cognitive decline in mild cognitive impairment for early prediction of progression to dementia of the Alzheimer’s type
Source: Sci Rep. 2024 Jun 5;14:12906. doi: 10.1038/s41598-024-63301-7 (PMC11153588; doi:10.1038/s41598-024-63301-7)
Supplement: Supplementary file 1 — Supplementary Information. [file 41598_2024_63301_MOESM1_ESM.docx]

1. **Methods:**

**1.1 Neuroimaging Measures**

Hippocampal, Entorhinal Cortex, Occipital Lobe, Fusiform Gyrus, Whole Brain, and Ventricle (left and right ventricles combined) volume measures were obtained from the ADNIMERGE datasheet from the ADNI database (<https://adni.bitbucket.io/reference/adnimerge.html>). The ADNIMERGE dataset merges the key cognitive scores and clinical biomarkers from all aspects of the ADNI study into one table that spans all four stages - ADNI1, ADNIGO, ADNI2, and ADNI3 - of data acquisition. These volumetric measures in the ADNIMERGE table were computed using USCF protocols for FreeSurfer cortical parcellation and subcortical segmentation. The details of the USCF Freesurfer protocol for ADNI data can be found here: https://adni.bitbucket.io/reference/docs/UCSFFSX51/UCSF%20FreeSurfer%20Methods%20and%20QC_OFFICIAL.pdf

**1.2 Neuropsychological Measures**

The item-wise cognitive scores used in our study were extracted from the NEUROBAT dataset from ADNI. NEUROBAT contains data across multiple assessments of participants on a neuropsychological battery (<https://adni.bitbucket.io/reference/neurobat.html>). Continuous scores were retained for factor analysis to determine latent cognitive process scores (Section 2.7), and measures that were systematically unavailable for specific cohorts of ADNI acquisition were excluded. For example, the digit-span test and the category-naming vegetable tests were discontinued after ADNI1 and consequently excluded from our study. Based on these criteria, we retained 13 variables from the NEUROBAT dataset.

The 9 measures of episodic memory from the RAVLT task are described as follows – To begin with, a list of 15 words is read out to the participant, and the participant has to recall as many words as they can. There are 5 such trials performed consecutively, with the same list, and the participant’s performance on each trial is stored in a separate variable (*AVTOT1, AVTOT2, AVTOT3, AVTOT4, AVTOT5*). The participant is then presented with a new list of 15 words, and the number of items they can recall from this list is recorded (*AVTOTB*). Their ability to recall items from the original list following the reading out of the second list is also recorded (AVTOT6). The administrator finally measures the participant’s ability to recall items from the first list after a 30-minute delay (*AVDEL30MIN*), and their ability to recognize the first list of words from a given list of words (*AVDELTOT*).


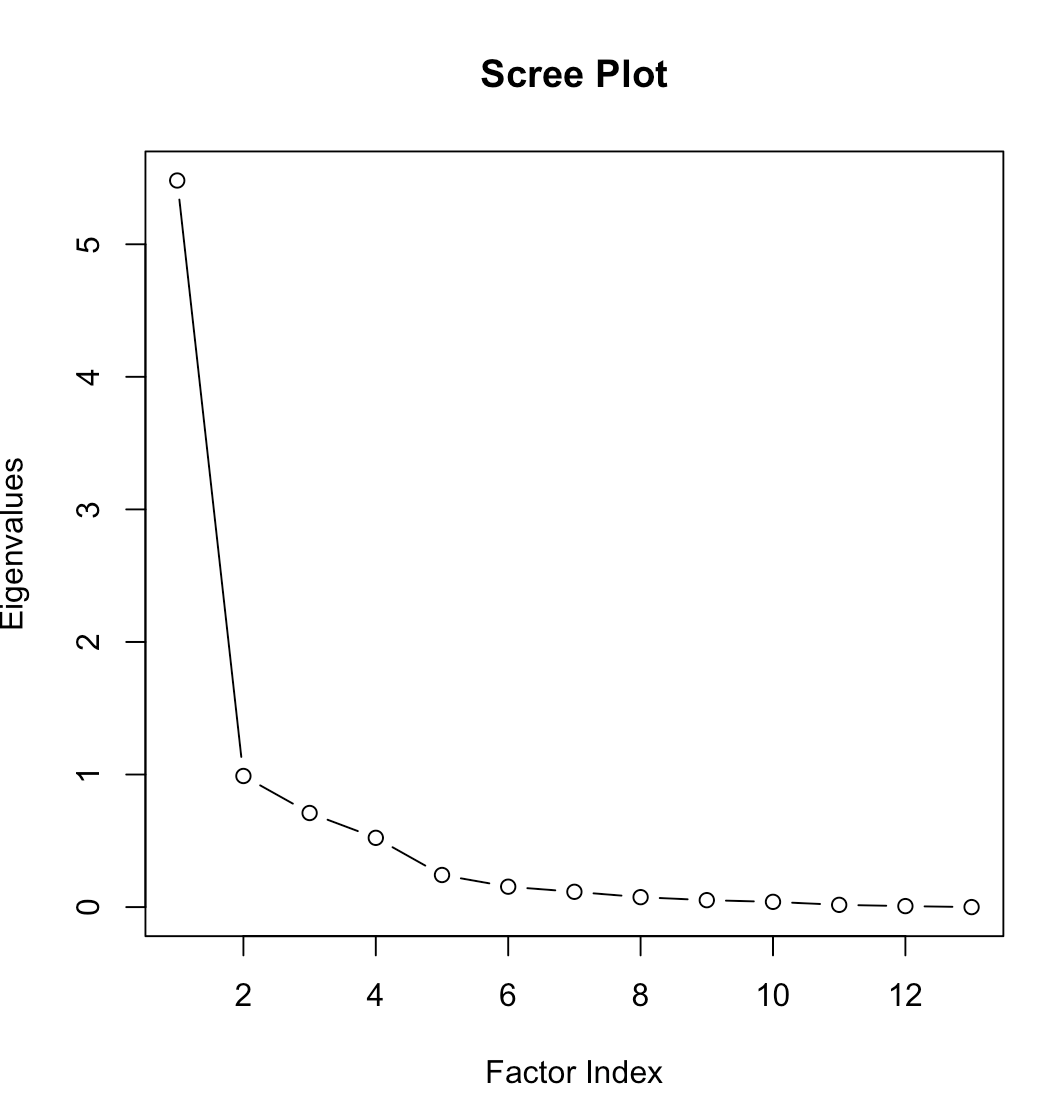


Figure S1: Relationship between Rates of Hippocampal and EC Volume Loss and Rates of Cognitive Decline – Factor Analysis


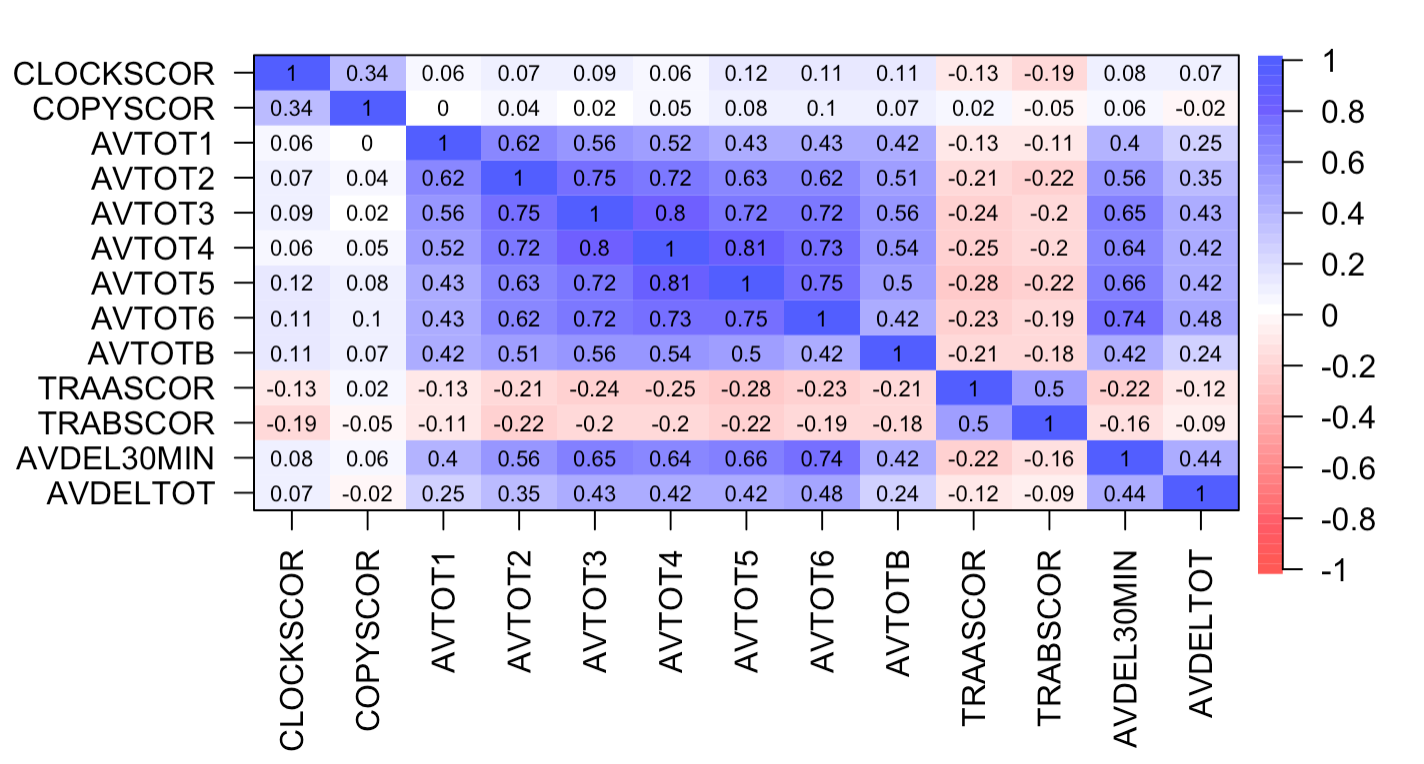


Figure S2: Correlation Matrix of Cognitive Scores from Cognitively Normal participants. In general, clock scores clustered together, the auditory and verbal learning scores clustered together, and the Trails scores clustered together.

All the AVLT scores seemed to load onto one factor from evaluating the correlation matrix. Save for the correlation between AVDELTOT and AVTOT1 (r =0.25 ), and AVDELTOT and AVTOTB (r =0.24), the pairwise correlations between AVLT scores were consistently above 0.3. Additionally, the pairwise correlations of AVLT scores with other cognitive scores were consistently below 0.3. Likewise, the trail-making test scores seemed to load onto a second factor and the clock scores seemed to load onto a third factor.

**Longitudinal Growth Curves of Regional Brain Volumes in the MCI Stage – Control Analyses**

Table S1: Predicting Standardized Fusiform Gyrus Volume from Age and Cohort

| Fixed Effects |  |  |  |  |  |
| --- | --- | --- | --- | --- | --- |
| Predictor | b | SE | df | t | p-value |
| Intercept | 0.21 | 0.05 | 613.94 | 4.19 | p<0.001 |
| Age | -0.41 | 0.04 | 410.11 | -10.4 | p<0.001 |
| Cohort | -0.65 | 0.07 | 610.89 | -9.17 | p<0.001 |
| Interaction | -0.02 | 0.07 | 522.87 | -0.38 | 0.705 |
| Random Effects | |  |  |  |  |
| Variation around intercept (u0j) | | 0.71 |  |  |  |
| Variation around slope (u1j) | | 0.17 |  |  |  |
| Correlation b/w random effects | | 0.02 |  |  |  |

Table S2: Predicting Standardized Occipital Lobe Volume from Age and Cohort

| Fixed Effects |  |  |  |  |  |
| --- | --- | --- | --- | --- | --- |
| Predictor | b | SE | df | t | p-value |
| Intercept | 0.11 | 0.05 | 613.31 | 2.28 | p<0.05 |
| Age | -0.3 | 0.04 | 374.37 | -7.72 | p<0.001 |
| Cohort | -0.32 | 0.07 | 619.49 | -4.31 | p<0.001 |
| Interaction | 0.1 | 0.07 | 449.54 | 1.44 | 0.15 |
| Random Effects | |  |  |  |  |
| Variation around intercept (u0j) | | 0.78 |  |  |  |
| Variation around slope (u1j) | | 0.11 |  |  |  |
| Correlation b/w random effects | | -0.35 |  |  |  |

Table S3: Predicting Standardized Ventricle Volume from Age and Cohort

| Fixed Effects |  |  |  |  |  |
| --- | --- | --- | --- | --- | --- |
| Predictor | b | SE | df | t | p-value |
| Intercept | -0.05 | 0.06 | 589.06 | -0.82 | 0.411 |
| Age | 0.74 | 0.03 | 437.07 | 22.04 | p<0.001 |
| Cohort | 0.35 | 0.09 | 599.03 | 4.08 | p<0.001 |
| Interaction | 0.28 | 0.05 | 566.45 | 5.12 | p<0.001 |
| Random Effects | |  |  |  |  |
| Variation around intercept (u0j) | | 1.14 |  |  |  |
| Variation around slope (u1j) | | 0.3 |  |  |  |
| Correlation b/w random effects | | 0.53 |  |  |  |

Table S4: Predicting Standardized Whole Brain Volume from Age and Cohort

| Fixed Effects |  |  |  |  |  |
| --- | --- | --- | --- | --- | --- |
| Predictor | b | SE | df | t | p-value |
| Intercept | 0.23 | 0.05 | 619.46 | 4.92 | p<0.001 |
| Age | -0.59 | 0.04 | 499.08 | -16 | p<0.001 |
| Cohort | -0.61 | 0.07 | 619.08 | -9.01 | p<0.001 |
| Interaction | -0.1 | 0.06 | 618.05 | -1.7 | 0.09 |
| Random Effects | |  |  |  |  |
| Variation around intercept (u0j) | | 0.64 |  |  |  |
| Variation around slope (u1j) | | 0.21 |  |  |  |
| Correlation b/w random effects | | 0.02 |  |  |  |


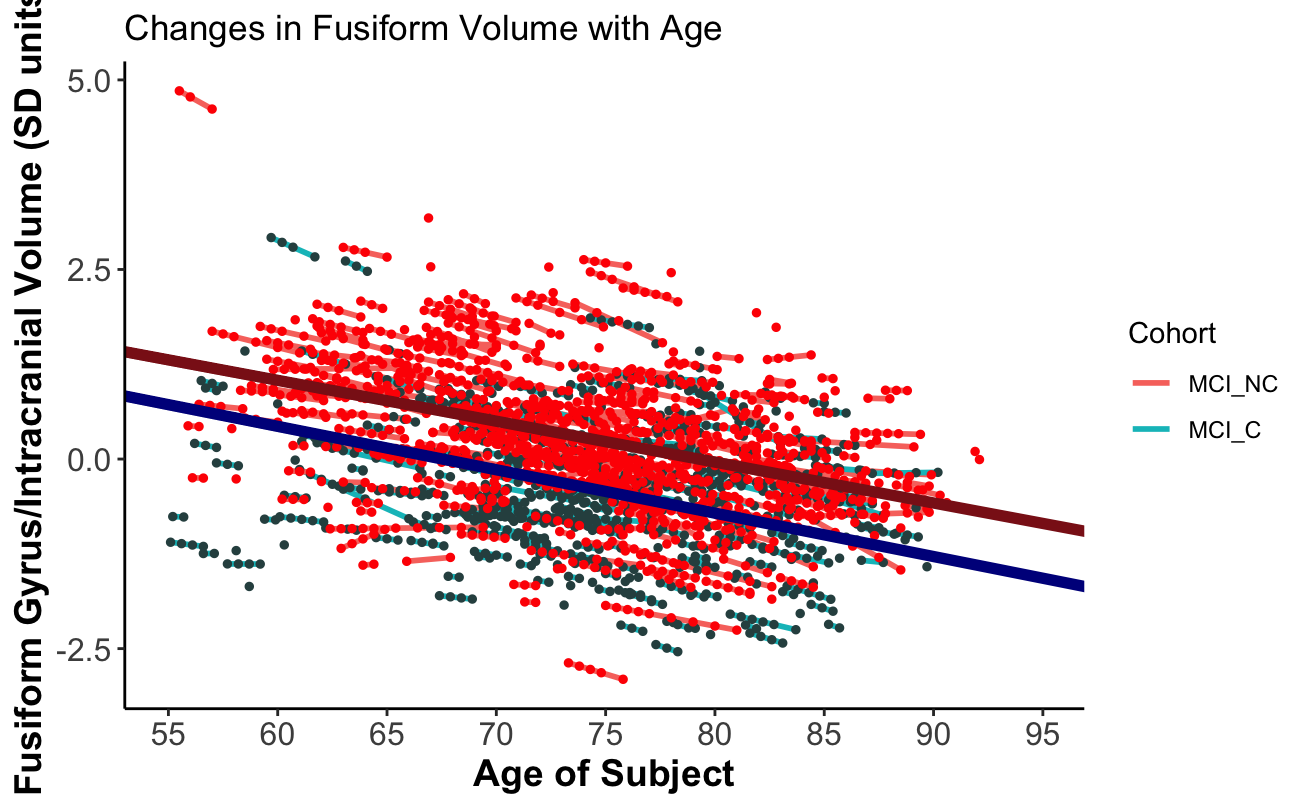


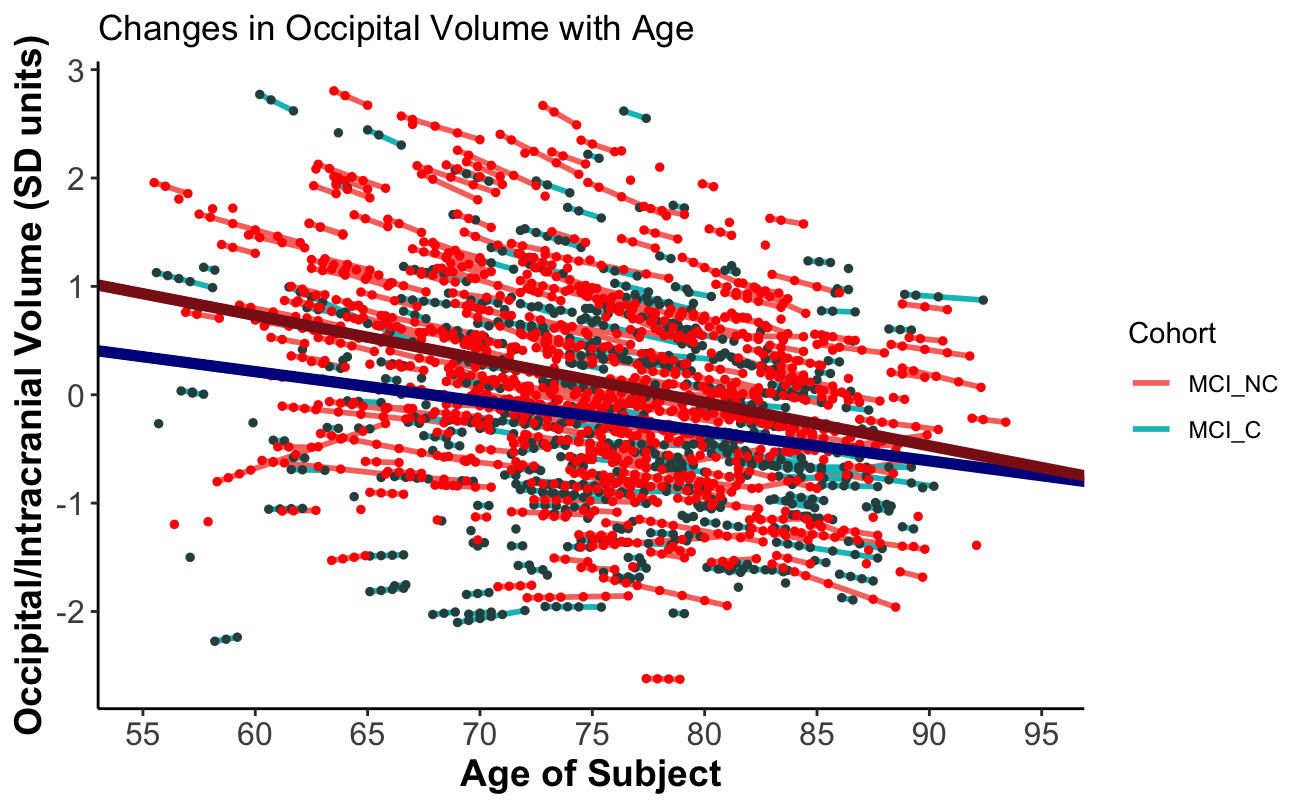


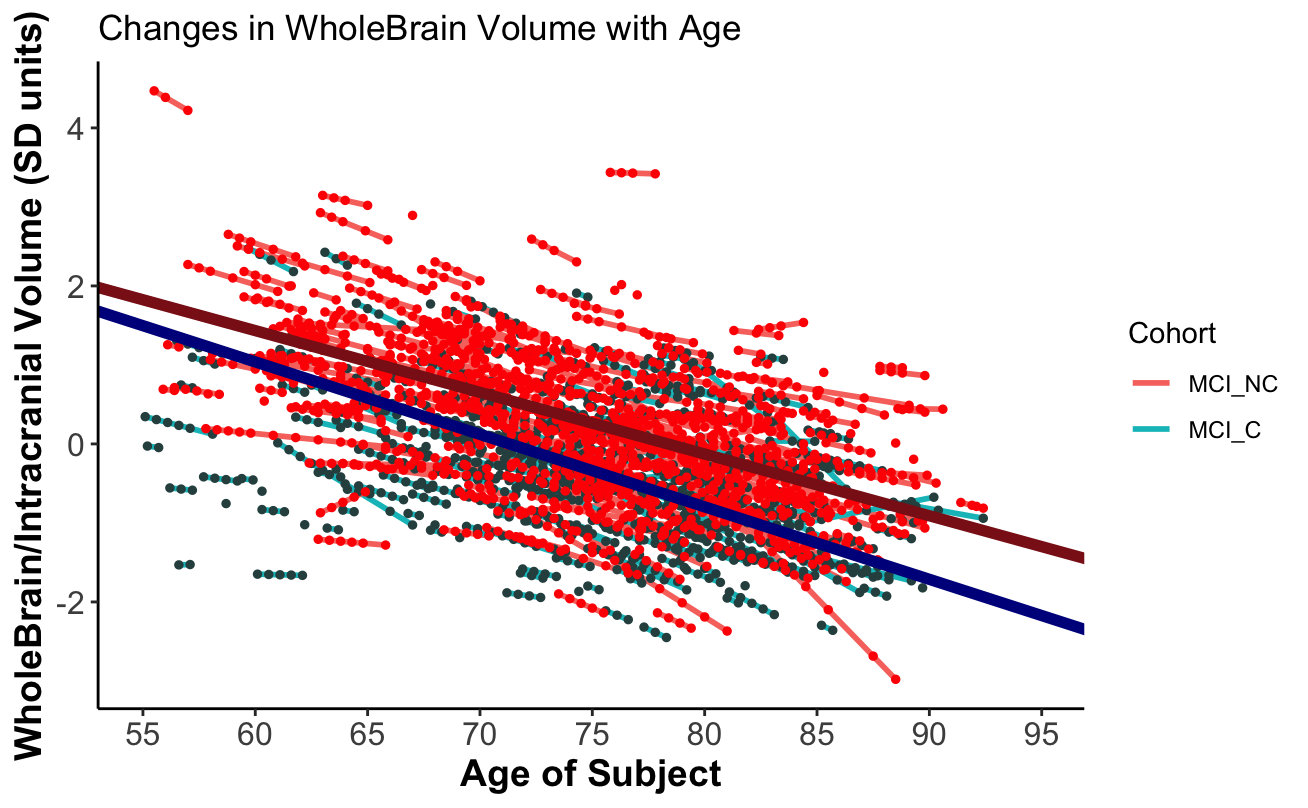


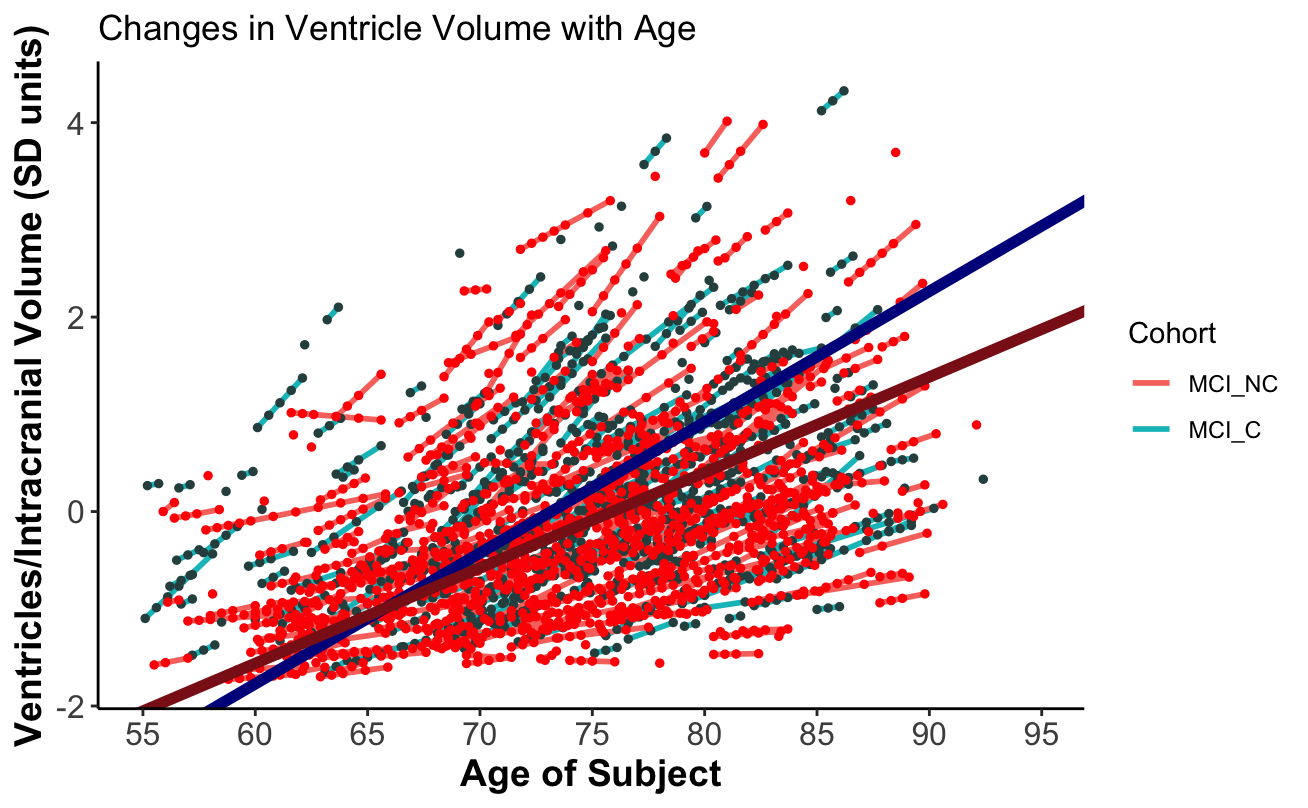


Figure S3: Growth Curve Models of Control Regions.

1. **Additional Analyses:**

2.1 Controlling for Difference in the Number of Longitudinal Followups across MCI Cohorts in Predicting EC Volumes

On average, MCI_C individuals have 3.5 repeat “MCI” visits, while MCI_NC have 6.02 repeat visits. To test if this was a possible alternative explanation for the lack of an interaction effect between age and cohort in predicting EC volumes, we repeated our analysis only including MCI_C individuals with 4 or more repeat visits (greater than average). We retained 149 MCI_C individuals with a new average of 5.48 repeat visits. Even with this data, we did not find a significant interaction between age and cohort in predicting EC volumes (b = -0.04, p =0.635).

2.2 Comparing an LME model to a Fixed-Effects Only model in Predicting hippocampal Volumes

To understand why a random slopes and intercepts LME model improved the fit and led to more robust effects in this case, it is helpful to look at the results of the fixed effects model, which is a simple linear regression model that does not account for any individual variation in either baseline values or rates of change as compared to the group average. The fixed effects model also found a significant interaction but with a smaller coefficient (b = 0.19 , p<0.001). While this linear regression model can still detect an interaction, it is detecting a weaker effect than the one that actually exists due to mistakenly including individual-specific variation as a part of the main effect. Additionally, this analysis deleted some observations (n = 1293) due to missing values. Finally, this analysis is not reliable since some of the assumptions of a linear regression are violated,including the independence and normality assumptions.

2.3 Repeating all Analyses in an Age-Matched Sample

To check if lower baseline measures of cognitive factors in MCI-Converters are due to the age of onset of MCI in Converters being a year later than Non-Converters on average, we matched the average age of onset of Non-Converters (M = 73.96) to Converters by iteratively removing 31 MCI-Non Converters in order of earliest age of onset. We repeated all our LME model analyses using this age-matched sample and found no change in our results. For example, even after age-matching, we find that MCI-Converters continue to have significantly lower baseline measures than MCI-Non Converters across all 3 cognitive measures, suggesting that a difference in the average age of onset cannot explain our findings
